# Patients’ Online Access to Their Primary Care Electronic Health Records and Linked Online Services: Implications for Research and Practice

**DOI:** 10.3390/jpm5040452

**Published:** 2015-12-04

**Authors:** Freda Mold, Simon de Lusignan

**Affiliations:** 1 School of Health Sciences, Faculty of Health and Medical Sciences, University of Surrey, Guildford GU2 7TE, UK; 2 Department of Health Care Management and Policy, University of Surrey, Guildford GU2 7XH, UK; E-Mail: s.lusignan@surrey.ac.uk

**Keywords:** internet, online systems, medical record systems, computerized, health care quality, health care quality, access, and evaluation, safety, general practice

## Abstract

Online access to medical records and linked services, including requesting repeat prescriptions and booking appointments, enables patients to personalize their access to care. However, online access creates opportunities and challenges for both health professionals and their patients, in practices and in research. The challenges for practice are the impact of online services on workload and the quality and safety of health care. Health professionals are concerned about the impact on workload, especially from email or other online enquiry systems, as well as risks to privacy. Patients report how online access provides a convenient means through which to access their health provider and may offer greater satisfaction if they get a timely response from a clinician. Online access and services may also result in unforeseen consequences and may change the nature of the patient-clinician interaction. Research challenges include: (1) Ensuring privacy, including how to control inappropriate carer and guardian access to medical records; (2) Whether online access to records improves patient safety and health outcomes; (3) Whether record access increases disparities across social classes and between genders; and (4) Improving efficiency. The challenges for practice are: (1) How to incorporate online access into clinical workflow; (2) The need for a business model to fund the additional time taken. Creating a sustainable business model for a safe, private, informative, more equitable online service is needed if online access to records is to be provided outside of pay-for-service systems.

## 1. Introduction

Providing online access to health care records and services may improve access and the personalization of primary health care. Online services have been described as fundamental to patient empowerment and may drive the improvement and organization of services [1,2].

Online access is the process of a patient, their carer, or their guardian logging on to access all or part of their electronic health record (EHR) with the ability to view, and sometimes edit or comment. Linked online services enable patients to communicate with their general practice, doctor, or other health care worker by email or through a web portal. Online services include tasks such as booking appointments or requesting repeat prescriptions (prescription refills) without seeing their doctor. Online access and services can be accessible from a patient’s home, workplace, or mobile computing device.

There have been some notable successes in the provision of online services in the United States. Organizations such as Kaiser Permanente and the Veterans Health Administration (VHA) have a large number of patients using their online services. These services include online appointment booking, repeat prescription requests, test result collection, and e-mail to clinicians [3,4].

In the UK, patient online access [5] has been successfully piloted [6], but not widely adopted beyond appointments and repeat prescriptions [7]. The successes seen in pilots of more extensive online services have yet to be more widely replicated. Progress to date has been limited by professional concerns about security and privacy [8,9,10], legal constraints [11], and low uptake [12]. These concerns are echoed in international studies [13]. Despite this slow progress, in 2010 the UK government announced in its health strategy that all patients in the English National Health Service (NHS) are to have access to their own health record by 2015 [14]. The guidance developed with the Royal College of General Practitioners (RCGP) was not widely adopted [15] and was subsequently revised [16].

This review aims to identify the key opportunities and challenges affecting online access to electronic health records and other online services in primary care.

## 2. Method

This review identifies new and recurring themes about online record access and services for research and practice. We updated our previous reviews with new evidence [1,2]. We replicated our previous search strategy, including hand searches, to ensure all additional evidence was identified between 2013 and 2015 [17]. We used a comprehensive search strategy/string comprised of a mixture of index terms (*i.e.*, MEDLINE/MeSH Terms) and keywords (in titles/abstracts). This search strategy/string is available by request. Similarly, a range of study designs were also included (qualitative, quantitative, mixed methods, and trial designs). Search results were stored using Endnote (v7). Evidence from 19 articles met our original inclusion/exclusion criteria. We excluded systems related to a single disease area, such as diabetes, as single disease conditions have developed their own applications with use driven by the needs of that specific condition.

## 3. Findings

### 3.1. Users and Non-Users of Online Access and Services

Adult females were the largest group of online access users [18,19,20,21,22,23,24,25,26,27,28] and were also the most active adopters of online services [20,22,23,24,25,26,27,28,29].

Unsurprisingly, lack of access to the internet was a barrier to use [30,31,32,33,34,35,36], though two studies reported it was not [37,38]. Patients reported being happy to share records with family or another health care professional and valued being able to print out segments of their records [18,30,39,40,41,42,43,44].

Previous studies have highlighted disparity in access between patient groups. There was lower use by non-white ethnicities [35,45], those from lower socioeconomic groups [33,46,47,48], and those in poorer health and from vulnerable groups [26,31,34,49].

### 3.2. Patient Safety

Online access may improve patient safety, primarily through identifying errors in medication lists and adverse drug reactions [26,39,50,51,52,53,54,55]. Identifying errors may limit the potential for severe harm [50].

There was no reported evidence of harm to patients from the provision of patient online access; though neither was there evidence of improved health outcomes [56,57,58]. Health professionals have reported concerns that viewing notes could potentially be disquieting to patients and might lead to the exploitation of the vulnerable. This in turn could impact negatively on patients’ willingness to disclose information and impact the doctor-patient relationship [18,30,39,59,60,61,62,63]. Recent studies have identified patients’ preference to control access to specific types of information, again raising concerns about patient safety if this information is not available to other health professionals involved in their care [64,65,66]. Patients wanted to choose the information they wish to share with family and their health care professionals, including the level of control based on their perception of the sensitivity of the record contents [66].

### 3.3. Patient Satisfaction

Patients who used online access reported positive experiences and satisfaction. They perceived these systems enabled better self-care [6,18,39,56,63,67,68,69,70,71,72,73] or empowered them to communicate more effectively with clinicians [39,40,41,58,59,63,69,73,74,75,76,77,78,79,80,81]. There was very little research reporting patients’ concerns about unauthorized access [81] or misuse [82].

Similarly, patients reported greater convenience, specifically time-saving compared with other methods of interaction with their health care provider [18,83,84,85,86,87,88,89,90]. Time-saving was also important to clinicians in terms of avoiding in-person clinic visits [86,87] and better management of patient care [91].

Satisfaction for patients depended on whether professionals were able to respond in a timely manner [25,47,53,62,83,92,93,94,95,96]. Differences emerged between patient and professional groups about whether online services (such as email contact) should be direct or not. Patients preferred direct communication with their health care provider [97,98,99,100], while clinicians preferred support staff to filter messages [45,61,100].

Email contact also had advantages for patients: they felt better able to express ideas or concerns [79,86,89,97,101,102,103,104], which again translated in to positive patient experience and greater satisfaction [25,50,57,71,77,78,85,89,95,97,98,105,106,107,108,109]. Little is known about the impact on health outcomes, and further research is needed in this area [110].

### 3.4. Workload and Organization of Care

Much of the research into online access and services suggested that clinicians are concerned about the potential effect on workload [111,112,113,114]. While several studies reported an increase in workload [21,32,39,100,115,116,117], other studies reported a large but temporary increase that plateaued in time [62,118]. Other studies described a decline in workload [57,62,63,81,86,116,117,119].

Studies report differing impacts on routine face-to-face consultations. Some report a decline in attendance [57,101,107,115,120,121], some an increase in attendance [21,39,118], and others no change [81,95,120,122]. Other forms of contact, such as email or web-messaging, may create a new and increased volume of contacts [57,78,107,115,116,117,118,123], while others report no change [89,97,108,124]. 

There was also an inconsistent impact on telephone contact; this may rise and then fall back when new services are offered [62,118]. Other studies reported no change in telephone volume [89,95,97,100,117,120], and a few described an increase [21,115,119].

There was little research of clinicians’ use of email to communicate with their patients; what research exists indicates that only a minority of clinicians (between 3% and 17%) regularly used email for this purpose [32,109,125,126,127]. Use of email to manage conditions was largely limited to problems that were manageable using this medium [24,25,34,79,83,88,94,106,115,118]. However, more complex problems were not suitable for this method of communication [88,99].

Online services have been perceived as fundamentally changing the organization of care, and implementation meant the re-organization of working practices [62,72,90,128]. Clinicians changed the way they wrote their medical records once they started to share these with the patient [63]. The nature of communication may also change. Changes included the tone, content, directness of the condition under discussion, and even a subtle shift in the balance of power in favor of the patient [61,92,103,114,129].

The rise of email appointment reminder systems in primary care decreased rates of failure to attend appointments [28]. The actual mode used to send the reminder was also important, some patients preferred email and others text messages [130].

### 3.5. Security and Privacy

Although patients expressed a willingness to trade-off security for ease of access [102,131], clinicians were more concerned, preferring proper controlled access that was more likely to protect patients’ privacy [55,132].

### 3.6. Fees for Services

Reimbursement to service providers seemed to be highly effective in promoting clinician uptake of online services. While some organizations in the USA (Kaiser and the VHA) have experimented with incorporating a fee, this practice is not widespread [87,133].

While many patients were positive about online services, they were not willing to pay for them. Patients who would consider a fee gave it a low financial value [31,34,93,134,135].

### 3.7. Technological Advancements

Research findings highlighted clinicians’ concerns about privacy and confidentiality [32,41,55,74,79,82,83,85,92,109,124,126,136,137,138,139]. In developing new contact systems, a formalized process is required to ensure governance procedures comply with existing regulations [43,45,90,94,102,109,118,125,128,140]. Although some clinicians lacked knowledge about what constituted an appropriate framework for protecting privacy [72,141,142], there is clearly a need for future guideline development [45,82,63,99,143,144,145]. More recent research focused on how to match technologies to different patient groups [146] and how to integrate these technologies into workflow [147].

A number of novel technologies had been introduced but not widely adopted:

- Links to X-ray and scan images [22,61,92];
- Automated tracking of test results [77];
- Text messaging question answering and answering machine services [140];
- Portals that can use codes or pictures of medications to avoid medication names being displayed [30];
- Web-based triage systems [24].

Integration of novel technologies required complex technical developments [61,92]. Many portals offering online access to records were designed to deliver full or partial online access [88,99]. However, despite this high level of technical innovation, levels of patient uptake remained low [23,24,43,70,100,139,148,149,150,151].

## 4. Implications for Research

### 4.1. Health Outcomes

Research into online access and services has yet to demonstrate how health outcomes can be improved. This may be particularly important for patients with complex needs, such as multiple comorbidities and associated polypharmacy. Research that evaluates the impact of online access in more complex cases compared to lower risk cases might provide some insight into where patient access and technology might add most value.

### 4.2. Organization of Care

Online access and services may challenge the established business process and organization of primary care:

- Computerized medical record systems may need to change to become more patient-friendly. This may, in the long term, enable patients to be more effective in self-management and involved in decision-making.
- Linking knowledge and information into online services may complement existing care in terms of changing the way clinicians communicate with patients and may indicate new ways to implement appointment reminder systems.
- Online access and services may change the nature of the patient-clinician interaction.
- Clinical and practice training may need to change to include effective communication; learning new styles and modes of communication.
- Clinicians also need to learn how it is possible to provide online access without being overwhelmed by online requests.
- Examination of users’ acceptance of online services and access, prior to implementation may provide insight into long-term sustainability.
- The re-design of services may need to be done so that it results in more accessible provision, which lessens current disparities.
- A business model that enables resources to follow the more efficient provision of additional online services.

### 4.3. Technological Advancement

Technological advancements need to incorporate the following:

- How the design of online record access may impact effective adoption and use of these technologies for different patient groups.
- How health care teams are best trained and assisted to support patients’ use of ever-changing technologies.
- How new systems can be integrated into the existing technological infrastructure and workflows.
- Whether these technologies are efficient and cost-effective.
- Whether the development of new systems can consider patient preferences, as different modes of contact (e.g., email) may alter user adoption and use. Ultimately, what circumstances and what forms of communication work best for patients and practitioners.
- Finally, although clinicians reported that ensuring privacy was of paramount importance, some patient evidence supported the view that they were willing to trade security for ease of access.

Although there were no reports of harm caused by breaches of privacy, concerns remain. Clinicians’ concerns about privacy might be mitigated by further guideline development. These new guidelines need to include in their scope the development of a common specification, which may reassure clinicians. These guidelines might then make computerized medical records more friendly and accessible to wider patient groups. Consideration also needs to be given to the functionality of systems, thereby enabling patients to have easy, but secure access.

There is a growing consumer demand to access (and possibly edit) data, and currently we have health care systems that are largely unprepared to meet these demands [152,153]. The promise of linking personal records from multiple sources into a readily digestible single online record makes this process potentially more complex [154,155].

### 4.4. Patient Safety

Online systems have the potential to improve patient safety. Current evidence is limited and needs to be strengthened. Further research needs to focus on:

- How patients use their access to computerized medical records and how this might enhance safety. To date, little is known about how online access and services are used for specific chronic and long-term conditions and how use will change with an aging population.
- How data added by patients to their records are used and the potential impact on patient safety.
- What is the evidence that online access and services increases or decreases patient anxiety, whether it fosters dependency, and what (if anything) needs to be done to mitigate this.
- The risk that important symptoms (e.g., chest pain) might be missed or not dealt with sufficiently urgently [156].
- Patients’ preferences over who can view their medical record; and the impact of such limitations on professionals’ ability to provide care and ensure patient safety.

## 5. Implications for Practice

Implementing online access and services have been shown to provide some opportunities; while there remain challenges for primary care practices, benefits include:

- Online access may positively impact the patient-physician relationship.
- Patients may find online services more convenient.
- Patients may express greater ownership over their medical record and be empowered.
- The ability to view medical records is no longer the sole provenance of clinicians; this alteration in ownership is likely to enhance relationships, overall.

However, there are also a range of possible negative consequences to offering online access and services. These include:

- Online access may make it easier for patients to always consult rather than to self-manage their conditions.
- Online access may result in variations in service utilization, with disparities of access for specific patient groups, and may increase disparities.
- Clinicians may need to be more careful about how they write records and not raise and share concerns, which form important prompts for colleagues.
- This is another skill and role for practice teams to acquire.
- New technologies may necessitate the need for on-going support and training of users, especially if adoption and use is to be sustained.

Further research is needed in four areas: health outcomes, organization of care, technological advancements, and patient safety.

Since our previous publications, technological innovations have advanced and new lessons have been learnt regarding the implementation of online access and services in primary care. New themes include patient added data, the possible use of online access for non-urgent cases, and patients’ preference to control access to specific types of information.

## 6. Conclusions

Online access offers patients more convenience and may improve patient safety and satisfaction. The USA has had some success in implementing online services. However, this is within a different and clearly defined business model for the delivery of these services. This success, and creation of an appropriate business model, has yet to be replicated in the UK. Explanations of low uptake beyond appointment booking, appointment reminders, and repeat prescription requests by UK patients, and lack of enthusiasm by health care professionals has not helped. This may be grounded in the lack of high quality evidence available. Evidence is needed about how to incorporate online access into quality of care, or how online services might positively impact health outcomes. Regardless, online access is here to stay, and will grow over time.

In the UK there is a need for a changed business model that promotes the use of online services, with the goal that once implemented, this may fundamentally change the business process in primary care, empower patients, and result in safer practice. With careful development, these services may be successfully incorporated into the organization of primary care.
